# Synergistic antifungal interaction of *N*-(butylcarbamothioyl) benzamide and amphotericin B against *Cryptococcus neoformans*

**DOI:** 10.3389/fmicb.2023.1040671

**Published:** 2023-03-07

**Authors:** Gabriella Maria Andriani, Lais Fernanda de Almeida Spoladori, Marciéli Fabris, Priscila Goes Camargo, Patrícia Morais Lopes Pereira, Jussevania Pereira Santos, Guilherme Bartolomeu-Gonçalves, Lais Alonso, Cesar Armando Contreras Lancheros, Antonio Alonso, Celso Vataru Nakamura, Fernando Macedo, Phileno Pinge-Filho, Lucy Megumi Yamauchi, Marcelle de Lima Ferreira Bispo, Eliandro Reis Tavares, Sueli Fumie Yamada-Ogatta

**Affiliations:** ^1^Programa de Pós-graduação em Microbiologia, Departamento de Microbiologia, Centro de Ciências Biológicas, Universidade Estadual de Londrina, Londrina, Paraná, Brazil; ^2^Laboratório de Biologia Molecular de Microrganismos, Departamento de Microbiologia, Centro de Ciências Biológicas, Universidade Estadual de Londrina, Londrina, Paraná, Brazil; ^3^Laboratório de Síntese de Moléculas Medicinais, Departamento de Química, Centro de Ciências Exatas, Universidade Estadual de Londrina, Londrina, Paraná, Brazil; ^4^Programa de Pós-graduação em Fisiopatologia Clínica e Laboratorial, Departamento de Patología, Análises Clínicas e Toxicológicas, Centro de Ciências da Saúde, Universidade Estadual de Londrina, Londrina, Paraná, Brazil; ^5^Instituto de Física, Universidade Federal de Goiás, Goiânia, Goiás, Brazil; ^6^Laboratório de Inovação Tecnológica no Desenvolvimento de Fármacos e Cosméticos, Departamento de Ciências Básicas da Saúde, Centro de Ciências da Saúde, Universidade Estadual de Maringá, Maringá, Paraná, Brazil; ^7^Laboratório de Imunopatologia Experimental, Departamento de Ciências Patológicas, Centro de Ciências Biológicas, Universidade Estadual de Londrina, Londrina, Paraná, Brazil

**Keywords:** antibiofilm, antivirulence, cryptococcosis, synergism, thiourea, urease

## Abstract

**Introduction:**

*Cryptococcus neoformans* is one of the leading causes of invasive fungal infections worldwide. Cryptococcal meningoencephalitis is the main challenge of antifungal therapy due to high morbidity and mortality rates, especially in low- and middle-income countries. This can be partly attributed to the lack of specific diagnosis difficulty accessing treatment, antifungal resistance and antifungal toxicity.

**Methods:**

In the present study, the effect of the synthetic thiourea derivative *N*-(butylcarbamothioyl) benzamide (BTU-01), alone and combined with amphotericin B (AmB), was evaluated in planktonic and sessile (biofilm) cells of *C. neoformans*.

**Results:**

BTU-01 alone exhibited a fungistatic activity with minimal inhibitory concentrations (MICs) ranging from 31.25 to 62.5 μg/mL for planktonic cells; and sessile MICs ranging from 125.0 to 1000.0 μg/mL. BTU-01 caused a concentration-dependent inhibitory activity on cryptococcal urease and did not interfere with plasma membrane fluidity. Molecular docking was performed on *Canavalia ensiformis* urease, and BTU-01 showed relevant interactions with the enzyme. The combination of BTU-01 and AmB exhibited synergistic fungicidal activity against planktonic and sessile cells of *C. neoformans.* Microscopic analysis of *C. neoformans* treated with BTU-01, alone or combined with AmB, revealed a reduction in cell and capsule sizes, changes in the morphology of planktonic cells; a significant decrease in the number of cells within the biofilm; and absence of exopolymeric matrix surrounding the sessile cells. Neither hemolytic activity nor cytotoxicity to mammalian cells was detected for BTU-01, alone or combined with AmB, at concentrations that exhibited antifungal activity. BTU-01 also displayed drug-likeness properties.

**Conclusion:**

These results indicate the potential of BTU-01, for the development of new strategies for controlling *C. neoformans* infections.

## 1. Introduction

Fungal diseases pose significant threats to global public health (Rodrigues and Nosanchuk, 2020). It is estimated that nearly 1.5 million people die yearly due to fungal infections (Bongomin et al., 2017). Among the fungal species that cause disease in humans, *Cryptococcus* spp. represent one of the most significant threats to human health. The global burden of cryptococcal meningitis is estimated at 152,000 cases (ranging from 111,000 to 185,000) annually, causing over 112,000 deaths (ranging from 79,000 to 134,000) in individuals living with human immunodeficiency virus (HIV) and approximately 19% of all acquired immunodeficiency syndrome-associated deaths (Rajasingham et al., 2022).

More than 30 species have been described within the genus *Cryptococcus.* However, few are recognized for their ability to infect humans (Findley et al., 2009; Maziarz and Perfect, 2016). In fact, most cryptococcal infections are caused by two closely related basidiomycetous species: *Cryptococcus neoformans* and *Cryptococcus gattii* complexes (do Carmo et al., 2022; Rathore et al., 2022). Cryptococcal infection begins in the respiratory tract after inhalation of basidiospores or dried yeast cells, from environmental reservoirs. In the lungs, the infectious propagules deposited in alveoli can be cleared by the host immune system or survive and establish a latent pulmonary infection. Nonetheless, the fungal cells can disseminate from the lungs, causing disease in several host tissues, mainly in the central nervous system (CNS), and less frequently, eyes, lymph nodes, bone, and skin (Gushiken et al., 2021). A significant proportion of infections are caused by *C. neoformans*, which mainly affects immunocompromised hosts, with meningitis being the most prevalent clinical condition (Cogliati, 2013; do Carmo et al., 2022).

Currently, there is no approved antifungal vaccine against invasive fungal infections (Biswas, 2021). Therefore, the antifungal agents remain as the only measure for the etiologic control of cryptococcal diseases. The polyene amphotericin B (AmB), whose discovery dates to 1953 (Carolus et al., 2020), is the first line for the treatment of cryptococcal meningitis, while the liposomal AmB is the preferred formulation recommended by the World Health Organization (WHO, 2018). Indeed, the standard protocol for treating this disease, especially in people living with HIV, is based on the induction, consolidation, and maintenance regimens. Induction therapy is aimed at decreasing the fungal burden in the host. Thus, intravenous AmB is used in combination with oral flucytosine for at least 2 weeks. This regimen is highly effective and reduces the risk of treatment failure and mortality compared to other regimens (Mourad and Perfect, 2018; WHO, 2018; Loyse et al., 2019; Iyer et al., 2021). Fluconazole monotherapy is associated with slower fungal clearance, higher relapse and mortality rates, and a greater predisposition to select resistant strains and, therefore, should only be used in the consolidation and maintenance phases (WHO, 2018; Iyer et al., 2021). Although AmB-resistant *C. neoformans* isolates are still rare (Carolus et al., 2020), the high price and unavailability of liposomal AmB and flucytosine in several countries (especially in low- and middle-income countries) limit the use of the induction regimen (Loyse et al., 2019; Rodrigues and Nosanchuk, 2020). Other concerns related to the use of AmB in the clinic involve its intravenous administration, which requires hospitalization and monitoring of blood counts and renal functions; and its toxicity, such as fever, nausea, and renal toxicity that may lead to discontinuation of treatment (Loyse et al., 2019; Carolus et al., 2020). Besides, the growth mode as a biofilm requires concentrations of the antifungal that are usually toxic to the host compared to their planktonic counterparts (Martinez and Casadevall, 2006; Tavares et al., 2019). For this reason, to reduce the overall incidence and mortality of cryptococcal meningitis, there is a severe need to develop effective and safe strategies for its treatment.

Thiourea is a class of organic compounds containing the > NC = SN < moiety that allows many chemical modifications and bonding possibilities due to the presence of nitrogen and sulfur atoms (Zahra et al., 2022). Several biological activities have been attributed to thiourea derivatives, including antibacterial, antifungal, antitrypanosomal, antidiabetic, antitumor, and urease inhibitor (Rego et al., 2018; Brito et al., 2020; Khidre and Radini, 2021; Pereira et al., 2021; Biasi-Garbin et al., 2022; Zahra et al., 2022) among others. Thiourea derivatives are thermally stable and can be produced with good efficiency from available starting compounds by simple and low-cost reactions (Shakeel, 2016). Therefore, in our ongoing search to discover new antimicrobial agents, in a previous study, we evaluated the antifungal activity of a series of benzoylthioureas against *Candida* spp. These compounds exhibited a moderate inhibitory activity on the growth of planktonic cells, with minimal inhibitory concentration (MIC) ranging from 250 to >1,000 μg/mL (Biasi-Garbin et al., 2022).

Encouraged by these preliminary results, in the present study, we report for the first time the antifungal activity of *N*-(butylcarbamothioyl) benzamide (BTU-01), a synthetic thiourea derivative, alone and combined with AmB, on planktonic and biofilm cells of *C. neoformans*. Furthermore, the effect of BTU-01, alone or combined with AmB, on some virulence attributes of this fungal species and viability of mammalian cells was also evaluated.

## 2. Materials and methods

### 2.1. Microorganisms and culture conditions

*Cryptococcus neoformans* ATCC 34872 (serotype A), *C. neoformans* ATCC 66031 (serotype D), and four *C. neoformans* human isolates (Table 1) belonging to the microbial collection of the Laboratório de Biologia Molecular de Microrganismos of Universidade Estadual de Londrina (UEL), Londrina, Paraná, Brazil, were included in the present study. Yeasts were cultivated in Sabouraud dextrose (SD) agar at 37°C for 48 h and stored at 4°C. For the experiments, five colonies were transferred to SD broth and incubated under the same conditions. Cells were then centrifuged (10,000 × *g* for 1 min) and resuspended in 0.85% NaCl solution (saline) to achieve turbidity equivalent to 0.5 McFarland standard using the DensiCHEK™ PLUS colorimeter (bioMérieux), which corresponds to approximately 1.0–2.0 × 10^6^ colony forming units (CFUs)/mL (standard fungal suspension). The fungal suspension was diluted in a culture medium to achieve the inoculum used in each assay. The study protocol was in accordance with the ethics committee of the UEL. The patients signed a written informed consent form agreeing with this publication (CAAE number 57452322.2.0000.5231).

**TABLE 1:** Minimal inhibitory concentration (MIC) and minimal fungicidal concentration (MFC) of *N*-(butylcarbamothioyl) benzamide (BTU-01) and amphotericin B for *Cryptococcus neoformans*.

Fungi	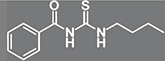 BTU-01		Amphotericin B	
	MIC^a^	MFC^b^	MIC^a^	MFC^b^
*C. neoformans* ATCC 34872	62.5	125.0	0.250	0.5
*C. neoformans* ATCC 66031	62.5	125.0	0.125	0.5
*C. neoformans* 1172	62.5	125.0	0.250	0.5
*C. neoformans* 90889	62.5	250.0	0.250	0.5
*C. neoformans* CN01	62.5	500.0	0.125	0.5
*C. neoformans* CN12	31.25	>1000.0	0.125	0.5

^a^MIC was determined as the lowest concentration capable of inhibiting the visual growth of fungal cells.

^b^MFC was determined as the lowest concentration capable of reducing the CFU counts to zero.

The results were determined after 72 h of incubation and were expressed in μg/mL.

### 2.2. Synthetic thiourea derivative and amphotericin B

The *N*-(butylcarbamothioyl) benzamide (BTU-01) was synthesized and characterized according to Brito et al. (2015). For all antifungal assays, BTU-01 was dissolved in dimethylsulfoxide (DMSO) to obtain a 10 mg/mL stock solution and further diluted in the culture medium to obtain the concentrations used in each assay. Stock solution of AmB (1.6 mg/mL; Merck, Brazil) was dissolved in 10% DMSO solution in ultrapure water and maintained at −20°C. DMSO did not exceed 1% in all assays.

### 2.3. Antifungal susceptibility testing on planktonic cells

Minimal inhibitory concentrations (MIC) of BTU-01 and AmB were determined by the broth microdilution method (CLSI, 2017). An inoculum (100 μL) of yeast cells (0.5–2.5 × 10^3^ CFU/mL) was added into the wells of a U-bottom 96-well microtiter plate (Techno Plastic Products, Switzerland) containing twofold serial dilutions of BTU-01 (1.95–1000.0 μg/mL) and AmB (0.031–16.0 μg/mL) in RPMI-MOPS. Wells containing medium or medium *plus* DMSO 1% with or without the fungal cells were used as growth and sterility controls, respectively. *Candida parapsilosis* ATCC 22019 was used as quality control. MIC was defined as the lowest concentration capable of inhibiting visual growth after 72 h of incubation at 37°C in comparison to growth control. The results of AmB were interpreted using the epidemiological cut-off values proposed in Espinel-Ingroff et al. (2012). To determine the minimal fungicidal concentration (MFC), 10 μL from the wells without visible growth was transferred onto SD agar (Espinel-Ingroff et al., 2002), and incubated at 37°C for 48 h. The MFC was determined as the lowest concentration capable of reducing the CFU counts to zero.

### 2.4. Effect of BTU-01 on the fungal plasma membrane

Electron paramagnetic resonance (EPR) spectroscopy of spin label was used to characterize the interactions of BTU-01 with the plasma membrane of *C. neoformans*. Fungal cells (2.0 × 10^8^ CFU/mL) were incubated in 2 mL of SD broth containing different concentrations of BTU-01 (5xMIC; 10xMIC; 20xMIC; and 20xMFC) under stirring at 37°C for 48 h. Subsequently, the cells were centrifuged (2,000 × *g*, 10 min) to reduce the final volume to 50 μL. To label the fungal membrane, spin-label film was first prepared on the bottom of a glass test tube using 1 μL of an ethanolic solution containing the lipid spin label 5-doxyl stearic acid (5-DSA, 2.0 mg/mL). After evaporation of the solvent, 50 μL of fungal suspension was added to the spin-label film, and the mixture was gently agitated. For the EPR analysis, each sample was transferred to a capillary tube (1 mm in diameter), which was sealed using a flame. EPR spectra were recorded using an EPR EMX-Plus spectrometer (Bruker, Germany) operating with the following instrument settings: microwave power, 2 mW; microwave frequency, 9.45 GHz; modulation frequency, 100 kHz; modulation amplitude, 1.0 G; magnetic field sweep, 100 G; scan time, 168 s; and sample temperature, 25 ± 1°C. The best fit EPR spectra were obtained using *non*-linear least squares software (NLLS) developed by Budil et al. (1996). As in previous studies, the rotational Brownian diffusion rate, Rbar, provided by the NLLS tuning program, was converted to the rotational correlation time parameter, *f*c, by the relation *f*c = 1/6 Rbar and was measured in nanosecond (Mendanha and Alonso, 2015; Alonso et al., 2020). The EPR spectra were simulated using one or two spectral component model with the same input parameters for g and A magnetic tensors: g_*xx*_(1) = 2.0086; g_*yy*_(1) = 2.0058; g_*zz*_(1) = 2.0025; A_*xx*_(1) = 6.3; A_*yy*_(1) = 6.3; A_*zz*_(1) = 32.0; g_*xx*_(2) = 2.0073; g_*yy*_(2) = 2.0058; g_*zz*_(2) = 2.0025; A_*xx*_(2) = 6.3; A_*yy*_(2) = 6.3; and A_*zz*_(2) = 32.0. The numbers (1) and (2) refer to the first and second spectral components, respectively. The average value of *f*c for each spectrum was calculated by the weighted average of the values of *f*c for the two components (*f*c1 and *f*c2), provided by the NLLS software, using the equation: *f*c = (f1*fc1 + f2*fc2), where f1 and f2 are the fractions of components 1 and 2 in the simulated spectrum.

### 2.5. Effect of BTU-01 on urease activity

The urease activity of *C. neoformans* was first screened on Christensen’s urea agar, incubated at 37°C for 48 h (Kurtzman et al., 2011).

#### 2.5.1. Crude protein extract preparation for urease assays

Five colonies of *C. neoformans* were suspended in 100 μL SD broth and inoculated onto SD agar using a Drigalski spreader, and the culture was incubated at 37°C for 48 h. Afterward, all fungal growth was gently scraped and transferred to conical flasks containing 300 mL of SD broth and incubated with shaking (150 rpm) at 37°C for further 48 h. Cells were centrifuged (6,000 × *g*, 10 min) and washed three times with distilled water. The protein extraction was performed according to Moller et al. (2016) with modifications. Briefly, fungal cells were resuspended in 10 mL of chilled lysis buffer consisting of 100 μM *N*α-tosyl-*L*-lysine chloromethyl ketone hydrochloride (TLCK), 1 mM EDTA, 10% glycerol and 0.15 M phosphate-buffered saline (PBS), pH 8.0. About 15 sterile glass beads (4–5 mm) were added to the suspension and the cells were lysed by vigorous mixing on a Vortex Genie^®^ 2 (Scientific Industries Inc., USA) for a total of seven cycles, each consisting of mixing for 30 s, followed by 1 min of cooling on ice. Then, the cell debris was removed by centrifugation (6,000 × *g*, 15 min, 4°C), and the protein content of the resulting supernatant was quantified using the Indophenol method (Weatherburn, 1967).

#### 2.5.2. Urease activity inhibition test

The inhibition of urease activity was evaluated by the indophenol method (Weatherburn, 1967). Thus, in 1 mL cuvettes, the reaction mixture was prepared by adding 220 μL of phosphate buffer solution (100 mM, pH 8.0), 400 μL of 10 mM urea (Merck, Brazil), 100 μL of crude protein extract and 40 μL of different concentrations of BTU-01 (15.0, 30.0, 45.0, 62.5, 95.0, 125.0, and 250.0 μg/mL). After incubation at 45°C for 10 min, 160 μL of phenol reagent (a mixture of 1% phenol and 0.05% sodium nitroprusside) and 160 μL of the alkaline reagent (0.5% NaOH and 0.1% sodium hypochlorite) were added in each cuvette. The reaction mixtures were incubated for further 10 min at 45°C, and the optical density (OD) was measured at 630 nm. Urease inhibition percentage (I%) was calculated according to the equation: I% = 100–(OD_BTU–01_/OD_control_) × 100. Thiourea was used as a standard urease inhibitor (Song et al., 2022). The assay was performed in triplicate, and the lowest concentration of BTU-01 capable of reducing 50% of the urease activity (Inhibitory Concentration–IC_50_) was determined by *non*-linear regression using GraphPad Prism 8.0.2.

### 2.6. Molecular docking

The crystallographic structure of *Canavalia ensiformis* urease was obtained from Protein Data Bank with code (PDB ID: 4H9M). The structure has a ligand co-crystalized in the active site, acetohydroxamic acid (AHA). In the BIOVIA Discovery Studio Visualizer program (v.21.1.0.20298) (Systèmes, 2016), the cofactors (EDO) present in the structure were deleted, and the conformer of Arg439 was removed. The geometry optimization (MM2 force field) and the 3D structure of BTU-01 were performed using Chemdraw (Evans, 2014).

The molecular docking simulations were executed with Genetic Optimization for Ligand Docking (GOLD, v.2022.2.0) (Jones et al., 1997). The binding site was defined as a 15 Å radius from the midpoint between the nickel atoms [Ni(901) and Ni(902)], with coordinates defined as *X* = 18.7825, *Y* = −57.8095, and *Z* = −24.1515. The redocking calculation was performed in flexible mode, with 50 anchoring poses for AHA, with the four scoring functions available in the GOLD software, GoldScore (Verdonk et al., 2003), ChemScore (Eldridge et al., 1997), CHEMPLP (Korb et al., 2007), and Astex Statistical Potential (ASP) (Mooij and Verdonk, 2005), and other settings were defined as default. The Root Mean Square Deviation (RMSD) defined the best scoring function. The smallest RMSD value obtained among the score functions available in the program was considered the best calculation. ASP scoring function was the one that obtained the lowest RMSD value, with 0.2875 Å for the AHA.

So, we used ASP (Mooij and Verdonk, 2005) as a scoring function, and the same protocol applied to AHA was used for the BTU-01 ligand. The highest score was used to determine the best conformation for docking analysis, and the intermolecular interactions were analyzed using the BIOVIA Discovery Studio Visualizer program. The figures were generated with the PyMOL Molecular Graphics System, Version 2.0.7, Schrödinger, LLC.

### 2.7. Checkerboard microdilution assay

The antifungal effect of BTU-01 combined with AmB was evaluated using the checkerboard broth microdilution assay, according to Scott et al. (1995). Twofold serial dilutions of BTU-01 (0.03–1000.0 μg/mL) and AmB (0.000001–16.0 μg/mL) were respectively added across the rows and columns of the U-bottom 96-well microtiter plates. Thereafter, 100 μL (0.5–2.5 × 10^3^ CFU/mL) of fungal inoculum was added and the plates were incubated at 37°C for 72 h. The Fractional Inhibitory Concentration (FIC) of each compound was determined by the ratio of the MIC obtained when the compounds were tested in combination and the MIC of the compounds tested individually. The FIC Index (FICI) was calculated from the sum of the FIC_BTU–01_ and FIC_AmB_, and the value was interpreted as: synergism FICI ≤ 0.5, no interaction 0.5 < FICI < 4.0, and antagonism FICI > 4.0 (Odds, 2003).

### 2.8. Characterization of the combined antifungal effect between BTU-01 and amphotericin B on planktonic cells

For all assays, fungal cells were incubated in RPMI-MOPS (except when specified) supplemented with BTU-01 and AmB alone at MIC and MFC values or combined at the synergistic MIC values. Untreated fungal cells were considered as control.

#### 2.8.1. Time-kill kinetics

The time-kill assay (CLSI, 1999) was performed to analyze the rate of fungal killing in the presence of the compounds alone or combined. The fungal cells (1.0 × 10^3^ CFU/mL) were added to the wells of U-bottom 96-wells microtiter plates containing RPMI-MOPS supplemented with BTU-01 and/or AmB and were incubated at 37°C. At determined time points (0, 24, and 48 h), 10 μL was removed from each well and 10-fold serial diluted in saline. A 10-μL aliquot of each dilution was inoculated onto SD agar and the CFU counts were carried out after incubation at 37°C for 48 h. Averaged data were plotted as log_10_ CFU/mL vs. time (h). The fungicidal effect of the compounds was defined as a 99.9% (3 log_10_) reduction in CFU/mL of the starting inoculum (Klepser et al., 1998).

#### 2.8.2. Fungal cell viability

Planktonic cell viability was evaluated using the LIVE/DEAD™ Yeast Viability Kit (Molecular Probes, Invitrogen) according to the manufacturer’s recommendations. Fungal cells (1.0 × 10^7^ CFU/mL) of *C. neoformans* were treated with the compounds at 37°C for 12 h. Subsequently, the cells were incubated with FUN™-1 and Calcofluor White™ MR2 dyes and examined by fluorescence microscopy (Zeiss Axio Imager 2) using fluorescein filters with excitation of 480 nm and emission ≥530 nm.

#### 2.8.3. Fungal capsule size

*Cryptococcus neoformans* was cultivated in a minimal capsular induction medium (15 mM glucose, 10 mM MgSO_4_, 29.4 mM KH_2_PO_4_, 13 mM glycine and 3 μM thiamine-HCl, pH 5.5) at 37°C for 7 days (Frases et al., 2009). Afterward, a standard fungal suspension was prepared as described above, and the cells (1.0 × 10^7^ CFU/mL) were treated with the compounds at 37°C for 48 h. Cells were collected by centrifugation (10,000 × *g*, 1 min), suspended in an equal volume of Chinese ink on glass slides, spread the smear evenly with coverslips, and observed with a Zeiss Axio Imager 2 microscope (Zeiss, Germany). The capsule size of 100 randomly chosen cells was measured by using the software ImageJ 1.49v.^1^ The capsule thickness was determined according to Spadari et al. (2019), which consisted of the difference between the diameter of the cell, including the capsule, with the diameter of the body cell within the cell wall.

### 2.9. Antifungal susceptibility testing on sessile cells

#### 2.9.1. Biofilm formation

The capacity of *C. neoformans* strains to form biofilm was evaluated on flat-bottomed 96-well polystyrene plates (Techno Plastic Products, Switzerland), as described by Martinez and Casadevall (2006). Thus, the biofilms were formed in SD broth statically at 37°C for 24, 48, 72, 96, and 120 h, with an inoculum of 1.0 × 10^7^ CFU/mL. After each incubation period, the viability of sessile cells was evaluated using the 3-(4,5-dimethylthiazol-2-yl)-2,5-diphenyltetrazolium bromide (MTT, Merck, Brazil) reduction assay according to the manufacturer’s recommendations. The assays were carried out in quintuplicate and performed on two separate occasions.

The 72-h biofilms of *C. neoformans* were analyzed using confocal laser scanning microscopy (CLSM). The biofilms were formed on glass coverslips of 9 mm in diameter. The coverslips were immersed in wells of 24-well cell culture plates (Techno Plastic Products, Switzerland) containing 1.0 mL of SD broth, and then the biofilm was formed as described above. The biofilms were gently washed once with PBS, pH 7.2. Four μL of PBS containing concanavalin-A-Alexa Fluor 488 conjugate (ConA; 25 mg/mL, Invitrogen, USA) were added on the biofilm and the plates were incubated for 2 h at room temperature. The biofilms were examined using a confocal laser scanning microscope [Objective type: HC PL APO CS2 63x/1.40 oil; magnification: 63x; zoom: 1.03; numerical aperture: 1.40 (Leica Microsystems, Germany)] with the respective excitation and emission wavelengths of 498 and 528 nm, and intensity and gain of 14 and 714, respectively (Tavares et al., 2019).

#### 2.9.2. Antifungal activity

To evaluate the antifungal activity of the compounds on sessile cells, biofilms were formed for 72 h, washed with saline, and 200-μL aliquots of RPMI-MOPS containing different concentrations of BTU-01 (31.25–1000.0 μg/mL) and/or AmB (0.007–16.0 μg/mL) were added for determining the SMIC (Sessile Minimal Inhibitory Concentration). Untreated and treated biofilms were incubated at 37°C for further 24 h, and then washed with saline. The biofilm viability was analyzed by the MTT reduction assay as described above. The SMICs of BTU-01 and AmB alone and combined were determined by the lowest concentration capable of inhibiting 80% (SCIM_80_) of the viability of sessile cells compared to the untreated controls. The checkerboard assays were performed to analyze the effect of the combination with BTU-01 and AmB on 72-h biofilms. The results of the combination were interpreted using the FICI as described previously.

### 2.10. Scanning electron microscopy (SEM) analysis of biofilms

Morphological alterations provoked by BTU-01 alone and combined with AmB on *C. neoformans* biofilms were analyzed by SEM. The glass surface (0.5 cm^2^) was immersed in wells of 24-well cell culture plates containing 1.0 mL of SD broth, and then the biofilm was formed as described above. The 72-h biofilms were treated with BTU-01 SMIC_80_ alone or with AmB at a synergistic concentration at 37°C for 24 h. The biofilms were fixed with 2.5% (v/v) glutaraldehyde in 0.1 M sodium cacodylate buffer pH 7.2 at room temperature, dehydrated with serial ethanol washes (30, 50, 70, 80, 90, 95, and 100 %), critical point dried in CO_2_, coated with gold and observed in a FEI Quanta 250 scanning electron microscope.

### 2.11. Effect of BTU-01 and amphotericin B on mammalian cells

The effect of BTU-01 was analyzed on mammalian epithelial cell lines [HEp-2 CCL-23™ (ATCC), LLC-MK2, VERO CCL81 and HeLa (Merck, Brazil)], and the cytotoxicity was evaluated as described by Longhi et al. (2016) with modifications. Briefly, cells were cultured in Dulbecco’s Modified Eagle’s medium (DMEM) supplemented with 10% fetal bovine serum (Invitrogen, Brazil), 2 mM L-glutamine, 100 IU/mL penicillin, 100 μg/mL streptomycin, 1% tylosin in flat-bottomed 96-well microtiter plates, in 5% CO_2_ at 37°C for 48 h. Then, the medium was carefully removed and fresh medium containing twofold serially diluted BTU-01 (0.095–500.0 μg/mL) was added. The cells were incubated for further 24 h under the same conditions. Cell viability was analyzed by the MTT reduction assay according to the manufacturer’s recommendation. The concentration of the compound needed to inhibit the viable cells up to 50 and 90% by regression analysis corresponds to the 50% (CC_50_) and 90% (CC_90_) cytotoxic concentrations, respectively.

The cytotoxicity of BTU-01 (1.95–1000.0 μg/mL) alone and combined with AmB (0.015–16.0 μg/mL) was evaluated on human erythrocytes. Blood from a healthy donor was collected according to the Declaration of Helsinki principles, defibrinated and an erythrocytes suspension (3%) was prepared in 5% glycosylated saline. A total of 100 μL of the erythrocytes suspension was incubated for 3 h at 37°C in wells of U-bottom 96-well polystyrene plates containing different concentrations of the compounds. Wells without the compounds and with 1% Triton X-100 were used as negative and positive hemolysis controls, respectively. The OD of the supernatant was determined at 550 nm with a microtiter plate reader (Synergy HT, BioTek, USA). The percentage of hemolysis was calculated by comparison with the positive control (Izumi et al., 2012).

The concentration of BTU-01 capable of causing 90% of hemolysis/cytotoxicity was used to calculate the selectivity index (SI) using the following equation: SI = CC_90_/MIC.

The effect of BTU-01 combined with AmB was also analyzed on HEp-2 CCL-23™ (ATCC) epithelial cell line using the combination of twofold serially diluted BTU-01 (0.095–500.0 μg/mL) and AmB (0.0125–1.0 μg/mL). The cells were incubated for further 24 h, and cell viability was analyzed by the MTT reduction assay, as described above.

### 2.12. *In silico* drug-likeness and ADME predictions

The free online platform SwissADME was used to predict the Absorption, Distribution, Metabolism, and Elimination (ADME) properties of the BTU-01 compound, and also molecular descriptors related to Lipinski’s “Rule of Five” (Lipinski et al., 2012) and extensions.^2^

### 2.13. Statistical analysis

GraphPad PRISM version 8.0.2 software (GraphPad Software, San Diego, CA) was used for statistical analysis. Data of the antifungal effect of BTU-01 alone and combined with AmB on growth kinetics, cytotoxicity and capsule size were analyzed by Two Way ANOVA, while the effect on biofilm and urease were analyzed by One Way ANOVA. For all assays, *p* < 0.05 was considered significant.

## 3. Results and discussion

### 3.1. The *N*-(butylcarbamothioyl) benzamide (BTU-01) inhibited the growth of planktonic cells, displaying a fungistatic effect on *C. neoformans* strains

First, the antifungal activity of BTU-01 was analyzed on planktonic cells of all *C. neoformans* strains, and the MIC and MFC values are shown in Table 1. MICs of BTU-01 ranged from 31.25 to 62.5 μg/mL and MFCs ranged from 125.0 to >1000.0 μg/mL, indicating a fungistatic effect. Regarding AmB, the MIC values ranged from 0.125 to 0.250 μg/mL, and the MFC was 0.5 μg/mL for all strains, indicating that they were susceptible to this antifungal (Espinel-Ingroff et al., 2012). Among several studies with bioactive thiourea derivatives, few have reported an antifungal effect on *Cryptococcus* spp. In a high-throughput screening with 361,675 molecules, *N*-substituted benzothioureas with fungicidal activity against *C. neoformans* were identified; and the study highlighted that the thiourea moiety was crucial for its anticryptococcal activity (Hartland et al., 2016). This scaffold inhibits the fungal cell wall integrity (Hartland et al., 2016) and the secretory pathway (Beattie et al., 2020), which is required for many essential fungal processes (Barlowe and Miller, 2013), including virulence factors production (Yoneda and Doering, 2006). In fact, these substances inhibited the melanin and capsule synthesis (Beattie et al., 2020). Another study, described the antifungal activity of 1-(5-benzylsulfinyl-1,3,4-thiadiazol-2(3H)-lidene)-thiourea series against *C. neoformans* (MIC ranging from 4.0 to 7.0 μg/mL) and *C. albicans* (MIC ranging from 4.0 to 6.0 μg/mL). Specifically, the 1,3,4 thiadiazole and thiourea/urea moieties were essential for antifungal activity (Mannam et al., 2019).

Data from the literature reported that thiourea derivatives inhibited the urease activity of several organisms, including bacteria (Rego et al., 2018; Song et al., 2022). Cryptococcal urease can degrade urea from different sources (natural ecological niches and within the human host) to produce ammonia, a readily assimilable nitrogen source for fungal growth and survival. Especially under nutrient-limited conditions at 37°C, urease activity is higher compared to nutrient-rich conditions at 26°C, indicating that this enzyme is associated with the functioning of vital metabolic pathways of *C. neoformans* (Toplis et al., 2020).

Still, urease is one of the main virulence factors of *C. neoformans* (Almeida et al., 2015). It plays an important role during the fungal invasion of the CNS in mice by increasing the fungal cells sequestration into the brain microcapillaries and transmigration sites into the brain (Olszewski et al., 2004; Shi et al., 2010). The use of flurofamide, a urease inhibitor, reduces fungal transmigration to the brain and, consequently, organ infection, prolonging the survival of mice infected with the *C. neoformans* H99 strain (Shi et al., 2010). In addition, urease has been shown to increase phagolysosomal pH, contributing to the persistence of *C. neoformans* within macrophages and their spread by these host cells (Fu et al., 2018). These findings suggest that cryptococcal urease may be a potential target for the development of new antifungals to treat cryptococcal meningitis. In this regard, we aimed to determine whether the BTU-01 could act as a urease inhibitor.

**FIGURE 1:**
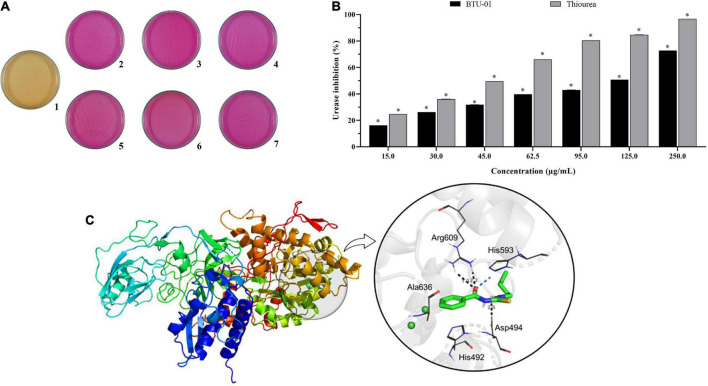
Effect *N*-(butylcarbamothioyl) benzamide (BTU-01) on urease activity of *Cryptococcus neoformans*. **(A)** Growth of *C. neoformans* in Christensen’s urea medium. (1) Absence of urease activity–negative control; (2) *C. neoformans* ATCC 34872; (3): *C. neoformans* ATCC 66031; (4): *C. neoformans* 1172; (5): *C. neoformans* 90889; (6): *C. neoformans* CN12; (7): *C. neoformans* CN01. **(B)** Percentage inhibition of BTU-01 on urease activity. Enzyme activity was evaluated by the indophenol method, and thiourea was used as urease inhibitor control. Values are mean ± standard deviation of three experiments. Asterisks indicate a significant inhibition activity in treated urease compared to untreated control (*p < 0.05). **(C)** BTU-01 interaction complex with urease (PDB: 4H9M). Ligand is in green carbon sticks and the amino acid residues are in gray carbon sticks. The other elements follow their colors: N: blue; O: red; H: white, and S: yellow. Conventional H-bonding is shown as black dots and unconventional H-bonding in marine dots.

All *C. neoformans* displayed the urease activity on Christensen medium (Figure 1A). As there was no significant difference (*p* > 0.05) among MIC values for all strains, *C. neoformans* ATCC 34872 was selected for this analysis. Thus, the inhibitory effect on cryptococcal urease activity was evaluated using the indophenol test, which is based on the reaction between ammonia and phenol to produce indophenol in the presence of an oxidizing agent and a catalyst (Weatherburn, 1967). Compared to the thiourea positive control, BTU-01 appears to be less potent as a urease inhibitor, according to IC_50_ values (BTU-01 = 53.3 μg/mL vs. thiourea = 38.6 μg/mL). Nevertheless, BTU-01 exhibited a concentration-dependent inhibitory activity of cryptococcal urease, as occurred to thiourea, with the percentage of inhibition ranging from 16.2 to 72.8% (Figure 1B).

To complete our study, we also investigate how the interaction between the BTU-01 with the urease enzyme could occur. Several studies indicate that fungal, plant and bacterial ureases have the same ancestor, since fungal and plant ureases have the highest similarity, as this functional unit is a single polypeptide chain (α), with a degree of identity above 50% (Balasubramanian and Ponnuraj, 2010; Kappaun et al., 2018). Due to the high similarity between the fungal and plant ureases, we use a urease from the plant *Canavalia ensiformis* (PDB ID: 4H9M) to analyze the protein-ligand complementarity through molecular docking studies. Firstly, the molecular docking protocol was validated by redocking in the GOLD program (Jones et al., 1997), using the ASP as a scoring function, with an RMSD of 0.2875 Å for the AHA.

The result of molecular docking simulations to BTU-01 showed that the *N*-carbamothiol moiety is, the fact, the most important scaffold present in the structure, responsible for the strongest interactions. The carbonyl group interacts through H-bond with the NHs of Arg609, at 2.2 and 3.0 Å of distance, and with CH of His593 through an unconventional H-bond (Figure 1C). Furthermore, the NH of the thiourea group of the BTU-01 does an H-bond at 2.6 Å of distance with the carbonyl group of Asp494, and the sulfur makes an electrostatic interaction, π–S type, with the imidazole ring of His593. As for hydrophobic interactions, we can see that the benzene ring of BTU-01 can interact with the imidazole ring of His492 through a π–π T-shaped stacked, and with Ala636 with a π– alkyl interaction. Finally, the butylamine moiety also makes a π– alkyl interaction with the imidazole of His593 (Figure 1C).

Although BTU-01 has shown a fungistatic effect, urease inhibition makes this compound attractive for developing new antivirulence agents, that is, substances that interfere with the pathogenicity mechanisms of the infectious agent (Vu et al., 2019).

### 3.2. BTU-01 exhibited a synergistic interaction with amphotericin B on planktonic cells of *C. neoformans*

**FIGURE 2:**
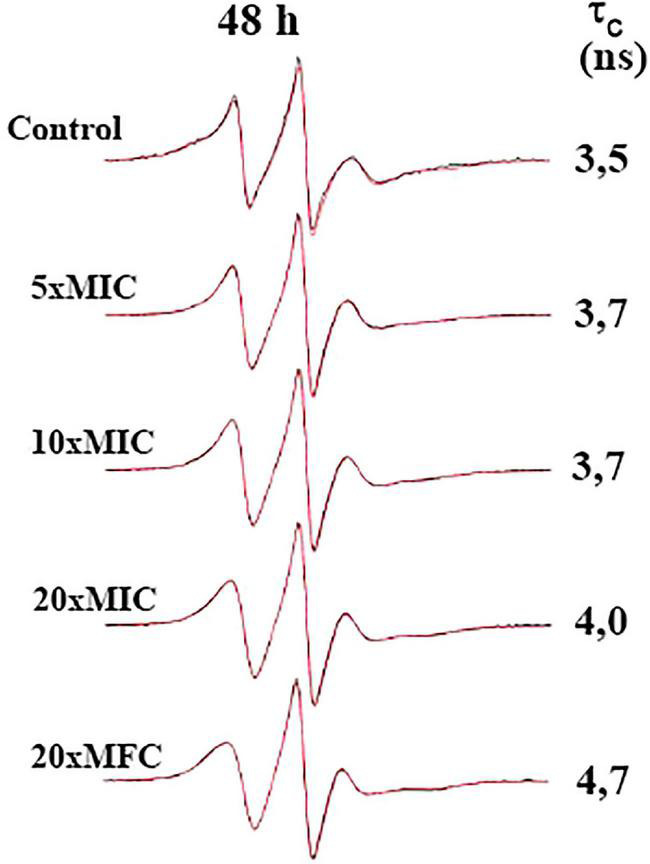
Effect of *N*-(butylcarbamothioyl) benzamide (BTU-01) on *Cryptococcus neoformans* plasma membrane fluidity. EPR experimental spectra (black) and best-fit (red) of 5-DSA spin label incorporated into plasma membrane of *C. neoformans* ATCC 34872 treated with different concentrations of BTU-01 (5xMIC; 10xMIC; 20xMIC; and 20xMFC) for 48 h. The simulated spectra (red) were provided by the NLLS program using a two-component spectral model. The rotational correlation time (*f*c) values provided by the simulation program were indicated. In all EPR spectra the total scan range of the magnetic field 100 G (*X*-axis) and the intensity (*Y*-axis) is in arbitrary units.

Due to the potential of BTU-01 as a urease inhibitor, we evaluated the *in vitro* effect of this thiourea derivative combined with AmB. The polyene antifungal targets the plasma membrane of fungal cells, and the best-known mechanism of its action is based on the transmembrane ion-channel model. In this sense, AmB-sterol complexes form ion-permeable channels in fungal cell membranes, leading to cell death (Kristanc et al., 2019). Initially, we evaluated whether BTU-01 could also target the cryptococcal plasma membrane, by EPR spectroscopy using the 5-DSA spin label. This lipid marker acts as a reporter that captures any slight change in the membrane in a *non*-destructive manner. The rotational correlation time (*f*c) indicates the time it takes the lipid marker to reset itself, that is, to turn around. The shorter this time, the greater the membrane fluidity (Camargos and Alonso, 2013). Compared to the untreated control cells, the EPR spectra of the 5-DSA inserted in the plasma membrane of *C. neoformans* ATCC 34872 did not cause any significant change in its dynamics after treatment with BTU-01, even at high concentrations. A small change in membrane fluidity was observed only in the presence of 20xMIC and 20xMFC values of BTU-01 (Figure 2). Alonso et al. (2022) showed that the treatment of the fungus *Paracoccidioides brasiliensis* with AmB caused a remarkable reduction in the spin label mobility, indicating an effect of membrane rigidity and/or an increase in the membrane polarity.

**FIGURE 3:**
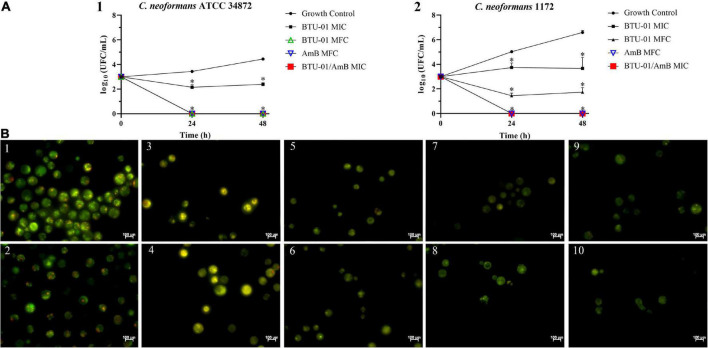
Synergistic antifungal interaction of *N*-(butylcarbamothioyl) benzamide (BTU-01) and amphotericin B (AmB) in *Cryptococcus neoformans*. **(A)** Time-kill kinetics of BTU-01, AmB and their synergistic combination. (1) *C. neoformans* ATCC 34872; (2) *C. neoformans* 1172. The log_10_ CFU/mL values were the mean and the standard deviation representative of three independent experiments. *p < 0.05. **(B)** Cell viability analysis of *C. neoformans* ATCC 34872 (1, 3, 5, 7, and 9) and *C. neoformans* 1172 (2, 4, 6, 8, and 10) after differential labeling with FUN™-1 dye. Yeasts were incubated with or without the MICs and MFC of the two compounds alone or combined for 12 h. Cells with diffuse greenish-yellow fluorescence characterize metabolically inactive cells and cells containing red fluorescent intravacuolar structures represent metabolically active yeast. (1 and 2) untreated viable cells; (3 and 4) BTU-01 MIC; (5 and 6) BTU-01 MFC; (7 and 8) AmB MIC; (9 and 10) BTU-01/AmB MIC.

Next, we evaluated the effect of BTU-01 combined with AmB on the growth of planktonic cells of *C. neoformans* ATCC 34872 and *C. neoformans* 1172 [serotype A, genotype VNI (Tavares et al., 2016); thereafter, these strains were named ATCC 34872 and 1172, respectively] by the checkerboard assay. There was a significant reduction in MIC values of BTU-01 and AmB in combination; both strains exhibited a 42-fold and 16-fold decrease in AmB and BTU-01 MIC values, respectively. The calculated FICI (0.08) indicated a synergistic antifungal interaction between BTU-01 and AmB (Table 2). The killing kinetics of *C. neoformans* planktonic cells in the presence of BTU-01 and/or AmB was monitored during 48-h incubation to analyze the rate and the nature of the synergistic interaction of both substances (Figures 3A1, 2). As expected, no CFU counts were detected in the presence of AmB MFC after 24 h-incubation. In the presence of BTU-01 MIC, there was an inhibition of growth over time and no significant change in the CFU counts compared to the initial inoculum, corroborating the fungistatic activity of BTU-01. However, after 48 h of incubation, differences of 2 log_10_ and 3 log_10_ in the CFU counts of ATCC 34872 (Figure 3A1) and 1172 (Figure 3A2) planktonic treated cells, respectively, were observed compared to the untreated control cells.

**TABLE 2:** Antifungal effect of *N*-(butylcarbamothioyl) benzamide (BTU-01) combined with amphotericin B (AmB) against planktonic cells and biofilms of *Cryptococcus neoformans*.

Microorganism	BTU-01	AmB	BTU-01/AmB	FICI	Interaction
Planktonic cells*					
*C. neoformans* ATCC 34872	62.5	0.25	3.9/0.003	0.08	Synergism
*C. neoformans* 1172	65.5	0.25	3.9/0.003	0.08	Synergism
72 h-biofilm**					
*C. neoformans* ATCC 34872	125.0	1.0	7.8/0.125	0.19	Synergism
*C. neoformans* 1172	1000.0	1.0	7.8/0.125	0.13	Synergism

*Minimal inhibitory concentrations were determined after 72 h of incubation.

**Biofilms were formed on polystyrene surface during 72 h at 37°C, and treated with different combinations of BTU-0 and AmB for further 24 h.

Minimal inhibitory concentration capable of reducing the viability of 80% of sessile cells (SMIC_80_). The values were expressed in μg/mL. FICI, fractional inhibitory concentration index.

Synergism–FICI ≤ 0.5; Antagonism–FICI ≥ 4 and Indifferent–FICI > 0.5–4 (Odds, 2003).

The treatment with MFCs led to zero CFU counts for ATCC 34872 (Figure 3A1), while for 1172 isolate (Figure 3A2), there was a significant reduction of 5 log CFU compared to the untreated control. On the other hand, BTU-01 combined with AmB reduced the CFU counts to zero for both strains after 24-h incubation, indicating a synergistic fungicidal effect.

The fungicidal effect of BTU-01 and AmB combined was further visualized by differential labeling using the fluorescent probes FUN™-1 and Calcofluor White™ MR2. Conversion of the fluorescent yellow-green stain of FUN™-1 into orange-red fluorescent intravacuolar structures requires plasma membrane integrity and metabolic capacity (Thermo Fisher Scientific, 2001). Thus, only metabolically active cells are labeled with intravacuolar fluorescent structures, as seen in untreated controls (Figures 3B1, 2). On the other hand, dead cells exhibit bright, diffuse yellow-green fluorescence as observed after the treatment with BTU-01 MFC (Figures 3B5, 6), AmB (Figures 3B7, 8) or at the synergistic concentrations of both compounds (Figures 3B9, 10). Cells with intact membranes but low or no metabolic activity exhibit diffuse green fluorescence and absence of intravacuolar fluorescent structure, as observed in yeast cells treated with BTU-01 MIC (Figures 3B3, 4).

**FIGURE 4:**
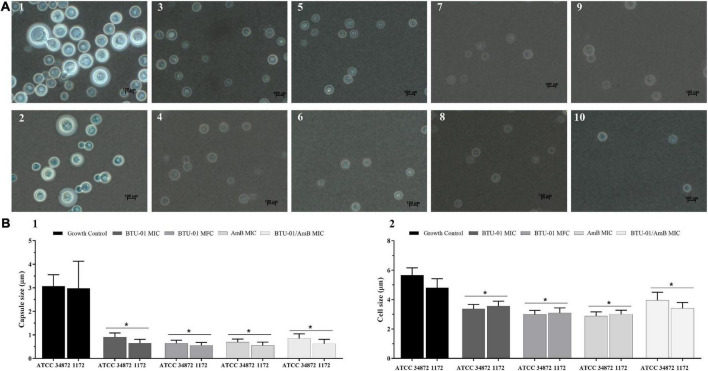
Effect of *N*-(butylcarbamothioyl) benzamide (BTU-01) and amphotericin B (AmB) alone or combined on capsule and cell sizes of *Cryptococcus neoformans.* **(A)** *C. neoformans* ATCC 34872 (1, 3, 5, 7, and 9) and *C. neoformans* 1172 (2, 4, 6, 8, and 10) were negatively stained with Chinese ink and visualized by light microscopy. (1 and 2) untreated cells; (3 and 4) BTU-01 MIC; (5 and 6) BTU-01 MFC; (7 and 8) AmB MFC; (9 and 10) BTU-01/AmB MIC. **(B)** The results of capsule (B1) and cell (B2) sizes were expressed in graphs and compared to the untreated controls. A total of 100 cells were measured and the mean ± standard deviation was calculated and analyzed by One Way ANOVA, *p < 0.05.

**FIGURE 5:**
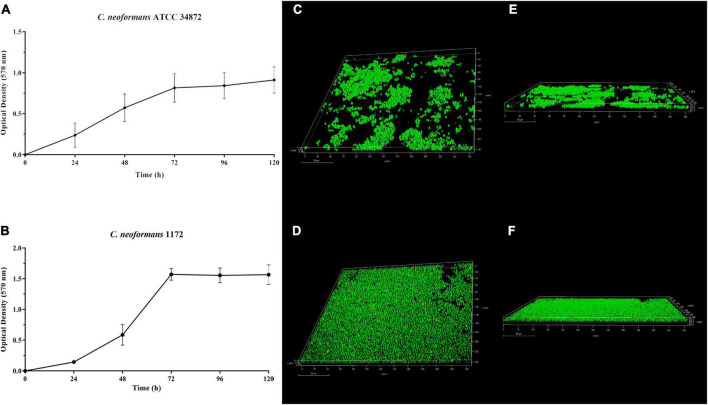
Temporal development of *Cryptococcus neoformans* ATCC 34872 **(A)** and *Cryptococcus neoformans* 1172 **(B)** biofilms on polystyrene surface monitored by measuring the metabolic activity of sessile cells using MTT reduction (OD_570 nm_) assay. The values represent the mean ± SD of three independent experiments. Confocal laser scanning microscopy (CLSM) images of the *C. neoformans* ATCC 34872 **(C,E)** and *C. neoformans* 1172 **(D,F)** biofilms formed on glass surface after 72 h at 37°C. **(C,D)** Panoramic view of biofilm; **(E,F)** Three-dimensional biofilm reconstitution.

**FIGURE 6:**
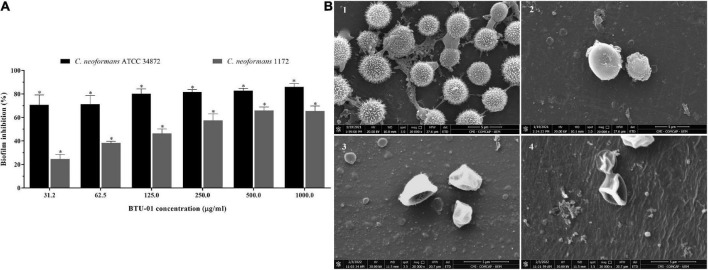
Antibiofilm activity of *N*-(butylcarbamothioyl) benzamide (BTU-01) in *Cryptococcus neoformans.* **(A)** Percentage of inhibition caused by different concentrations of BTU-01 on 72 h-biofilm of *C. neoformans* ATCC 34872 and *C. neoformans* 1172. Metabolic activity of sessile cells was assessed by the MTT reduction method. Values are mean ± standard deviation of three experiments. Asterisks indicate a significant reduction in the metabolic activity of treated sessile cells compared to untreated ones (*p < 0.05). **(B)** Scanning electron microscopy images of 72 h-biofilms of *C. neoformans* ATCC 34872 on glass surface. A total of 72 h-biofilms were treated with the compounds during 24 h at 37°C. (1) Untreated control biofilms; and after treatment with (2) BTU-01 SMIC; (3) AmB SMIC; (4) at synergistic concentration of BTU-01/AmB SMIC.

In the absence of effective monotherapy, combination therapies are being used clinically to treat potentially fatal invasive fungal infections (Chang et al., 2017; Iyer et al., 2021). Particularly for cryptococcal meningitis, fungicidal activity is an important property for effective antifungal therapy. Actually, AmB combined with flucytosine is considered the standard fungicidal treatment for cryptococcal meningitis (WHO, 2018), and is associated with improved survival of patients with cryptococcal meningitis compared to treatment with AmB monotherapy (Molloy et al., 2018; Loyse et al., 2019; Vidal et al., 2021). *In vitro* experiments also suggest that combinations of natural and/or synthetic compounds with available antifungals can improve the effectiveness of treatments and extend the efficacy of currently used antifungal agents (Andriani et al., 2021; Benelli et al., 2021; Iyer et al., 2021). The synergistic fungicidal interaction between BTU-01 and AmB may contribute to developing new strategies for controlling cryptococcosis meningitis. Notably, the fungicidal effect was obtained in lower concentrations of AmB, compared to the mono treatment with this antifungal.

### 3.3. BTU-01 combined with amphotericin B decreases the capsule size and inhibits the biofilms of *C. neoformans*

Polysaccharide capsule is anchored to the fungal cell wall, and its production is one of the main virulence factors of *C. neoformans*. Among its essential functions, the capsule contributes to both evasion of the immune defense and the survival of the fungus within the susceptible host (Zaragoza et al., 2009). In this sense, the study of Vélez et al. (2022) reported that the capsule size, which was determined under *in vitro* induction conditions and an infection model in *Galleria mellonella*, has been associated with the pathogenic potential of different clinical isolates of *C. neoformans* VNI. In addition, it has been shown that the capsule increases in size during the course of pulmonary infection in mice (Feldmesser et al., 2001; Zaragoza et al., 2005).

AmB has been reported to reduce the capsule size of *C. neoformans in vitro* (Nosanchuk et al., 1999) and in murine infection (Zaragoza et al., 2005). In this study, a significant reduction (*p* < 0.05) in capsule (Figures 4A, B1) and cell (Figures 4A, B2) sizes was observed after the treatment with BTU-01 (Figures 4A3–6, B1, 2) and AmB (Figures 4A7, 8, B1, 2) alone, compared to untreated cells of ATCC 34872 (Figures 4A1, B1, 2) and 1172 (Figures 4A2, B1, 2) cryptococcal strains. Although there was also a significant reduction (*p* < 0.05) in capsule size compared to untreated controls, no significant difference was observed for the combined treatment (Figures 4A9, 10, B1, 2) compared to the mono treatments.

The capsule is mainly composed of glucuronoxylomannan (GXM, around 90.0%) and two minor components galactoxylomannan (9.0–10.0%) and mannoproteins (<1.0%) (Cherniak and Sundstrom, 1994). These exopolysaccharides, especially GXM, have been associated with another crucial function of the capsule, which is its ability to promote the adhesion of *C. neoformans* to abiotic and biotic surfaces (Camacho and Casadevall, 2018). Contact with surfaces can trigger several cellular behaviors, including biofilm formation (Martinez and Casadevall, 2005). Cryptococcal biofilms protect against harsh conditions during saprophytic and pathogenic lifestyles (Martinez and Casadevall, 2005; Kernien et al., 2018). Clinically, biofilm-associated infections are challenging to eradicate due to their resistance to antifungal and host defenses (Kernien et al., 2018). Cryptococcal biofilms are resistant to azole antifungals (fluconazole and voriconazole); and although AmB and the echinocandin caspofungin can interfere with the release and extracellular accumulation of GXM, interrupting the formation of the exopolysaccharide matrix that is essential for biofilm formation, this effect is only achieved at concentrations higher than those found in the host (Martinez and Casadevall, 2006; Tavares et al., 2019).

In this study, both *C. neoformans* strains were capable of forming biofilms on polystyrene and glass surface (Figure 5). The temporal development of *C. neoformans* biofilms was assessed on polystyrene surface, and was monitored by measuring the metabolic activity of sessile cells (Figures 5A, B). There was a gradual increase in metabolic activity of sessile cells in the first 24 h. After that time, a substantial increase in the metabolic activity was observed, and although it remained high, a plateau was reached after 72 h (Figures 5A, B). *C. neoformans* 1172 formed a more extensive biofilm on glass surface (Figure 5D) compared to *C. neoformans* ATCC 34872 (Figure 5C). However, the morphological characteristics of both biofilms were similar. CLSM analyses showed a monolayer arrangement of cells firmly attached to the glass surface with about 8-μm-thick biofilms (Figures 5E, F).

The treatment of 72-h biofilms of *C. neoformans* with BTU-01 alone and combined with AmB caused significant inhibition of the metabolic activity of sessile cells in all concentrations tested. A percentage reduction ranging from 70.8 to 86.1% and 24.6 to 65.4% in sessile cell viability was observed for ATCC 34872 and 1172 strains, respectively. BTU-01 SMIC_80_ values of 125.0 and >1000.0 μg/ml were determined for ATCC 34872 and 1172, respectively (Figure 6A). For AmB, a SMIC_80_ of 1.0 μg/mL was identified for both strains. Previous studies have shown that the metabolic activity of mature biofilms of *C neoformans* ATCC 24067 (serotype D), ATCC B3501 (serotype D), and H99 (serotype A) strains was inhibited by AmB at concentrations higher than 2.0 μg/mL (Martinez and Casadevall, 2006). Unlike what was observed in this study, the biofilms of these strains were about 76-μm-thick, and this may explain the difference in SMIC values.

The combined treatment with BTU-01 and AmB significantly reduced the SMIC_80_ values of these compounds on 72-h biofilm for both strains (Table 2). There was a 16-fold and 8-fold reduction in SMIC_80_ values of BTU-01 and AmB, respectively, for ATCC 34872; while for 1172 strain, there was a 128-fold and 8-fold reduction of the SMIC_80_ of BTU-01 and AmB, respectively. The interaction of both compounds on biofilms was also classified as synergistic, with calculated FICI values of 0.19 and 0.13 for ATCC 34872 and 1172 strains, respectively (Table 2).

Untreated and treated biofilms of ATCC 34872 formed on a glass surface during 72 h were analyzed by SEM. The untreated control biofilms showed yeast cells with typical rounded morphology, displaying extracellular fibrils connecting the cells to each other and to the glass surface (Figure 6B1). In contrast, treatment with BTU-01 SMIC (Figure 6B2), AmB SMIC (Figure 6B3) and the combination of both (Figure 6B4) showed a significant decrease in the number of cells in the biofilm and the absence of extrapolymeric matrix surrounding the cells. Cellular damage, such as irregular shape and wilted cells, indicating leakage of cellular contents, was also observed in biofilms treated with AmB alone or in combination with BTU-01.

The potential of thiourea derivatives as antibiofilm agents on bacterial biofilms has been reported previously. For instance, thiourea derivatives bearing 3-amino-1H-1,2,4-triazole scaffold inhibited the biofilm formation of methicillin-susceptible and methicillin-resistant *Staphylococcus epidermidis* on polystyrene surface (Stefanska et al., 2016). Similarly, the S-3,4-dichlorobenzyl isothiourea hydrochloride inhibited the biofilm formation of multi-drug resistant *Pseudomonas aeruginosa* on polystyrene and polyethylene surfaces (Bonez et al., 2017). The 1,2,4-triazolyl-benzoylthiourea exhibited bacteriostatic activity against planktonic cells of *Staphylococcus aureus*, and could inhibit biofilm formation as well as eradicate the 6-h biofilm on polystyrene surface (Pinheiro et al., 2020). Finally, the combination of *N*-[2-(4-ethylphenoxy)methyl]-*N*-(arylcarbamothioyl)benzamides with Fe_3_O_4_ and polyvinylpyrrolidone for coating the silicon and glass catheter surfaces inhibited the adhesion and biofilm formation of *S. aureus* ATCC 25923 and *P. aeruginosa* ATCC 27853 on these medical devices (Limban et al., 2014).

### 3.4. BTU-01 combined with AmB does not cause toxicity to mammalian cells *in vitro*

The effect of new antifungal compounds on mammalian cells is of paramount importance since fungal cells display similarities with human cells (Campoy and Adrio, 2017). Thus, we initially evaluated the effect of BTU-01 on the viability of different cell lines and the CC_50_ (ranging from 61.0 to 121.2 μg/mL) and CC_90_ (ranging from 142.6 to 243.7 μg/mL) values are shown in Table 3. Considering the most frequent MIC value (62.5 μg/mL), SI values ranging from 2.1 to 3.9 were determined for BTU-01, indicating that this compound may be slightly more toxic to fungal cells.

**TABLE 3:** Effect of *N*-(butylcarbamothioyl) benzamide (BTU-01) on the viability of different cell lines, demonstrated by the cytotoxic concentrations (CC_50_, CC_90_), and the selectivity index (SI).

Cell line	CC_50_ μg/mL	CC_90_ μg/mL	SI
HeLa	83.8	153.7	2.5
HEp-2 CCL-23	104.2	245.0	3.9
LLC-MK2	71.7	142.6	2.3
VERO-CCL81	121.2	243.7	3.9

The hemolysis assay can be used as a model for human cells, as erythrocytes are considered sensitive to variations in the medium (Bonarska-Kujawa et al., 2015). BTU-01, alone and combined with AmB, did not show a significant hemolytic effect, nor did the treatment with AmB alone at concentrations with antifungal effect. The percentage of hemolysis for the tested concentrations ranged from 0 to 3.18% for BTU-01 and 0.52 to 1.34% for AmB. In view of this, it was not possible to calculate the CC_90_ of BTU-01 for human erythrocytes, so the highest concentration tested was used to estimate the SI, and values greater than 16 were detected for all cryptococcal strains, corroborating that BTU-01 may be more toxic toward the fungal species. The *non*-toxic activity of AmB in this study may be due to both the short incubation time (3 h) and the low concentration tested, since the hemolysis induced by AmB increases with the incubation time and treatment with 6 mg/mL of this antifungal induces cellular lysis (Butler et al., 1965; Szponarski and Bolard, 1987). Similarly, at synergistic concentration of BTU-01 combined with AmB, the percentage of hemolysis ranged from 0 to 3.02% (data not shown).

**FIGURE 7:**
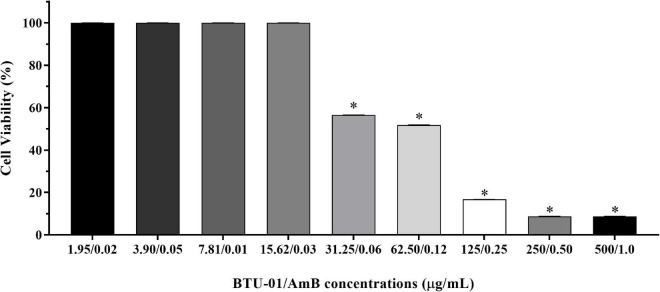
Effect *N*-(butylcarbamothioyl) benzamide (BTU-01) combined with amphotericin B (AmB) on mammalian cell viability. HEp-2 CCL-23 cells were treated with different concentration combinations of BTU-01 and AmB (1.95–500.0 μg/mL; 0.02–1.0 μg/mL, respectively). Cell viability was measured by the MTT reduction assay after 48 h of incubation at 37°C. Values are mean ± standard deviation of three experiments. Asterisks indicate a significant reduction in the metabolic activity of treated cells compared to untreated ones (*p < 0.05).

The effect of the BTU-01 combined with AmB was also evaluated in HEp-2 human epithelial cells line (Figure 7). By using the MTT reduction assay, around 51.8% of epithelial cells remained viable at the combination of BTU-01 (61.25 μg/mL) and AmB (0.12 μg/mL), indicating that the synergistic combination of both compounds (3.9 μg/mL BTU-01 and 0.003 μg/mL AmB) may be safe for the host.

### 3.5. *In silico* studies indicate drug-likeness, and good physicochemical and pharmacokinetic parameters of BTU-01

The *in silico* predictions were performed to analyze the drug-likeness-related physicochemical and pharmacokinetic properties of the BTU-01. According to Lipinski’s Rules (Lipinski et al., 2012), the following properties indicate that a compound have good solubility and membrane permeability profile when administered orally: clog*P* ≤ 5, H-bond acceptors (HBA) < 10, H-bond donor groups (HBD) < 5, and molecular weight (MW) ≤ 500 g/mol. Besides, Egan et al. (2000), Muegge (2003), Ghose et al. (2012), and Veber extension (Veber et al., 2002) expanded the correlations with absorption, distribution, metabolism, excretion, and toxicity (ADMET) parameters, which added important contributions to Lipinski’s rules.

Based on these parameters, BTU-01 may be a good candidate for an oral drug since the compound has all the similarities required according to Lipinski’s Rules and does not violate other drug-likeness rules. With HBA and HBD below ten and five H-bond, respectively; clogP of 2.64 and cLogS nearby −3.0; and MW of 236.33 g/mol, the compound is soluble to moderately soluble in water and has a good liposolubility. Other parameters that are related to absorption and permeation are the topological polar surface area (TPSA), which correlates substance transport with human intestinal absorption, and the number of rotatable bonds (NRB), which influences the permeation rate of the compound through membranes (Testa and Krämer, 2009); both parameters have adequate values for BTU-01, with TPSA of 73.22 Å2 and NRB = 7, indicating that the compound has good permeability to membranes.

Regarding pharmacokinetics, the compound is highly likely to be absorbed from the gastrointestinal tract; and it can also reach the CNS as it crosses the blood-brain barrier, which can be desirable for the treatment of cryptococcal meningitis. In addition, BTU-01 does not act as a substrate for P-glycoprotein but as an inhibitor of CYP1A2 and CYP2C19 isoenzymes, which could lead to adverse drug interactions and alteration in the activation, inactivation, and excretion of xenobiotics in the body (Zanger and Schwab, 2013).

## 4. Conclusion

In conclusion, the present study reports for the first time the antifungal and antivirulence activities of the *N*-(butylcarbamothioyl) benzamide (BTU-01), a synthetic thiourea derivative, on *C. neoformans*. Molecular docking indicates that the BTU-01 displayed strong interactions with key residues at the active site of urease (*C. ensiformis*), a relevant virulence factor of *Cryptococcus* spp., suggesting an inhibitory potential, which was corroborated by the *in vitro* results. Moreover, its combination with AmB exhibited a potent fungicidal and synergistic interaction against planktonic and sessile (biofilm) cells in concentrations that were not toxic to mammalian cells. Furthermore, *in silico* predictions showed good solubility and membrane permeability profiles, indicating the possibility of oral administration, which is an advantage compared to AmB. Limitations of this study, which may reduce generalization of the results, are: (a) the number of clinical isolates tested that do not represent all serotypes and genotypes of *C. neoformans.* In this study, most of the assays were performed with *C. neoformans* serotype A, genotype VNI strains, since it has been isolated more frequently in Brazil (Nishikawa et al., 2003; da Silva et al., 2020; do Carmo et al., 2022); (b) all tests were performed under *in vitro* conditions. Further studies aimed at evaluating the effectiveness of antifungal activity and toxicity *in vivo* are needed to corroborate the *in vitro* results; (c) and the mechanism of action has not been fully elucidated. Despite the limitations of this study, these results indicate that this thiourea derivative can be considered a promising prototype for the development of new strategies to control *C. neoformans* infections.

## Data availability statement

The original contributions presented in this study are included in the article/supplementary material, further inquiries can be directed to the corresponding author.

## Ethics statement

The studies involving human participants were reviewed and approved by the Ethics Committee of the Universidade Estadual de Londrina: CAAE number 57452322.2.0000.5231. The patients/participants provided their written informed consent to participate in this study.

## Author contributions

GA and SY-O performed the conception, experimental design, analysis and interpretation of data, and writing of the manuscript. All authors have read and approved the final manuscript, and have made a substantial methodological and intellectual contribution to the study.

## Footnotes

1
https://imagej.nih.gov/ij/

2
http://www.swissadme.ch
